# Using Consensus Bayesian Network to Model the Reactive Oxygen Species Regulatory Pathway

**DOI:** 10.1371/journal.pone.0056832

**Published:** 2013-02-15

**Authors:** Liangdong Hu, Limin Wang

**Affiliations:** Key Laboratory of Symbolic Computation and Knowledge Engineering of Ministry of Education, Jilin University, Changchun, Jilin, P. R. China; Queen's University Belfast, United Kingdom

## Abstract

Bayesian network is one of the most successful graph models for representing the reactive oxygen species regulatory pathway. With the increasing number of microarray measurements, it is possible to construct the Bayesian network from microarray data directly. Although large numbers of Bayesian network learning algorithms have been developed, when applying them to learn Bayesian networks from microarray data, the accuracies are low due to that the databases they used to learn Bayesian networks contain too few microarray data. In this paper, we propose a consensus Bayesian network which is constructed by combining Bayesian networks from relevant literatures and Bayesian networks learned from microarray data. It would have a higher accuracy than the Bayesian networks learned from one database. In the experiment, we validated the Bayesian network combination algorithm on several classic machine learning databases and used the consensus Bayesian network to model the 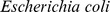
's ROS pathway.

## Introduction

Reactive Oxygen Species (ROS) are formed as by-products of normal metabolism of aerobic organisms, they can react with DNA and produce damage [1]. Cells protect themselves from ROS by detoxification mechanisms and repair mechanisms [2], [3]. Microarray is a powerful tool for measuring a large number of genes' expressions. Given the microarray expressions, it is possible to construct the regulatory pathway that organisms response to the oxidative stress directly.

An outstanding idea is the use of Bayesian network for representing regulatory pathway [4]–[7]. Bayesian network is a Directed Acyclic Graph (DAG) used for representing probabilistic relationships between variables. It was first proposed by Pearl [8], and Jensen [9] gave an intuitive definition. A lot of work has been done in the automatic learning of Bayesian network from database. Consequently, large numbers of Bayesian network learning algorithms based on different methodologies have been developed [10]–[13] and they have high accuracies in learning Bayesian networks from classic machine learning databases. However, when applying these algorithms to learn Bayesian networks from microarray data, the accuracies are low. Careful studies show that this is because the databases they used to learn Bayesian networks contain too few microarray data. On the other hand, microarray chip is expensive, it is difficult to obtain a large number of microarray data from one laboratory or one database, and a few hundred expression data can not guarantee a high learning accuracy.

To overcome this problem, we propose a consensus Bayesian network which is constructed by combining several Bayesian networks. This consensus Bayesian network is approximately equal to the Bayesian network learned from the database obtained by merging all these combined Bayesian networks' corresponding databases, then its equivalent database may have enough data and the accuracy can be improved. The main procedure of construction of consensus Bayesian network can be described as follow: (1) Review all relevant literatures and derive the Bayesian networks. (2) Search microarray expressions which are not used in relevant literatures and download them to learn Bayesian networks. (3) Combine all these Bayesian networks to construct the consensus Bayesian network.

Combination of Bayesian networks includes combination of graph models and aggregation of probability distributions [14]–[17]. Utz [18] proposed a method to combine many different Bayesian networks into an undirected graph, and each edge in the graph has a weight represents the frequency with which the edge occurs in the component networks. Zhang et al. [19] proposed a method for fusing Bayesian networks. They construct an initial network based on the union and intersection of the Bayesian networks, and then search for the structure which maximizes the scoring function(CH criterion). Our Bayesian network combination algorithm is based on the properties of probability. Due to probabilistic independence, Conditional Probability Tables (CPTs) can be extended, then corresponding nodes' CPTs can be changed into a same form and the aggregation function can be applied to these CPTs. After extending every corresponding CPTs, the combination of Bayesian networks changed into the aggregations of every corresponding nodes' CPTs if these Bayesian networks' variables' prior orders are consistent with each other. Some nodes' CPTs were extended previously, so they may have bogus parents after combination, then we should find them, delete the bogus edges and simplify the CPTs. The combination algorithm can also be applied to the combination of Bayesian networks defined over different variable sets by using the extension of Bayesian network.


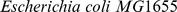
 was used in the experiment, a constructed ROS pathway was derived from the literature wrote by Hodges et al. [20] and 612 microarray expression data were downloaded from the Many Microbe Microarrays Database(M3D) [21]. 27 genes were identified from the EcoCyc [22] ROS detoxification pathway. A consensus Bayesian network using the 27 genes as variables was constructed by combining the Bayesian network from the literature and the Bayesian network learned from the 612 microarray expressions. For demonstrating the combination of Bayesian networks defined over different variable sets, we used a prediction program to find genes may be involved in the ROS pathway, learned a Bayesian network which using the 27 genes and the newly found genes as variables, and then combined this Bayesian network and the Bayesian network from the literature to construct a new consensus Bayesian network.

## Results

### Validation on classic machine learning databases

In order to validate whether the consensus Bayesian network 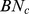 constructed by combining Bayesian networks 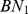 and 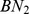 is equivalent to the Bayesian network learned from the database obtained by merging the two Bayesian networks' corresponding databases 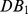 and 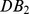 or not, 6 databases were downloaded from the UCI Machine Learning Repository (http://archive.ics.uci.edu/ml/), and the databases of ALARM net and Chest-clinic net were generated by the BN PowerConstructor. For each database 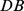, we chose 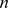 samples (about 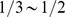 of the samples in 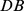) randomly and used them as 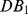, the rest samples in 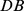 were used as 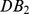. Two Bayesian networks 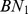 and 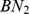 were learned from 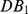 and 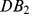, respectively. Consensus Bayesian network 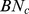 was constructed by combining 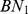 and 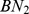. After that, another Bayesian network 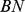 used as a reference was learned from 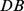, 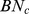 was compared with 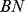 and the proportion of the number of identical edges between 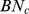 and 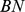 to the total number of edges in 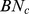 and 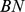 (similarity 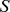) was computed. The program was run 100 times to compute the average similarity. All results of the experiments are shown in Table 1. Table 1 shows that all the average similarities are greater than 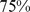. So, consensus Bayesian network 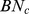 is approximately equal to Bayesian network 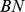. Although the combination algorithm is validated with 8 different databases and the types of data in these databases are very different, it doesn't affect the results. The Bayesian network learning algorithm just compute the distributions by counting the number of samples, and determine the relationships between the variables by analyzing the distributions. The Bayesian network combination algorithm is used to combine Bayesian networks and it doesn't involve the data. So, the type of data doesn't affect the validation.

**Table 1:** Validation of the combination algorithm.

Database			similarity	*T*(*s*)	similarity	*T*′(*s*)
*Letter Recognition*	17	20000	100.0%	0.000009	100.0%	0.000010
*Shuttle*	10	14500	100.0%	0.000008	100.0%	0.000008
*Parkinsons Telemonitoring*	26	5875	79.4±2.2%	0.086804	77.9±1.2%	1437.502573
*Image Segmentation*	20	2310	80.6±1.7%	0.066748	78.0±1.9%	835.820385
*Contraceptive Method Choice*	10	1473	83.2±2.1%	0.033214	82.5±2.5%	18.325412
*Solar Flare*	13	1389	75.0±3.0%	0.043424	76.6±2.5%	261.702598
*ALARM net*	37	10000	97.8±2.2%	0.123528	95.6±2.2%	372.952340
*Chest-clinic net*	8	1000	93.4±0.4%	0.026708	93.4±0.4%	12.259816

Where is the number of variables in the database, is the number of samples in the database, similarity is the average proportion of the number of identical edges between and to the total number of edges in and , and is the execution time of the Bayesian network combination program. The table shows that the similarity is depend on the number of samples, this is because the algorithms are based on the computation of probabilities and the accuracy of computation of probability is sensitive to the number of samples. Specifically, there are two reasons: (a)The real distributions of variables can't be reflected if the database only have several samples; (b)The equation we used to compute the probabilities is sensitive to the number of samples. Then in the experiments on and , similarity , this is because the two databases have enough samples and can provide enough information for constructing the real Bayesian networks, then the learned Bayesian networks , and are completely the same. So, consensus Bayesian network , and and are the same. Similarity and execution time are the results of the experiments using the fusion method proposed by Zhang et al. [19] instead of our combination algorithm. and show that our algorithm works more efficiently. The time complexity of our algorithm is , where is the number of nodes in the network. However, the execution time of Zhang's fusion method grows exponentially as the size of the biggest clique in the Clique tree increases.

Consensus Bayesian network 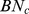 is approximately equal to Bayesian network 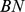, then we can view 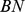
's database 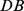 as 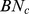
's equivalent database, and 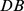 have more samples than 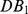 or 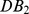. So, the use of consensus Bayesian network helps to solve the problem of lack of data in partial databases and the accuracy can be improved. The true structures of ALARM net and Chest-clinic net are known. Then we compared the learned networks with the known networks, the results are shown in Table 2. Table 2 shows that 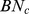 has a higher accuracy than 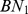 or 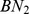.

**Table 2:** Comparison of the accuracies.

		ALARM			Chest-clinic	
	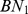	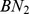	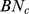	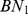	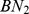	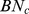
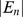	52	49	48	10	12	8
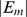	0	1	0	1	0	0
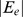	6	4	2	3	4	0

Where 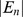 is the number of edges in the Bayesian network, 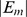 is the number of missing edges, 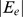 is the number of extra edges. The true structures of ALARM net and Chest-clinic net contain 46 directed edges and 8 directed edges, respectively.

### Construction of consensus Bayesian network for modeling 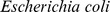
's ROS pathway

**Figure 1:**
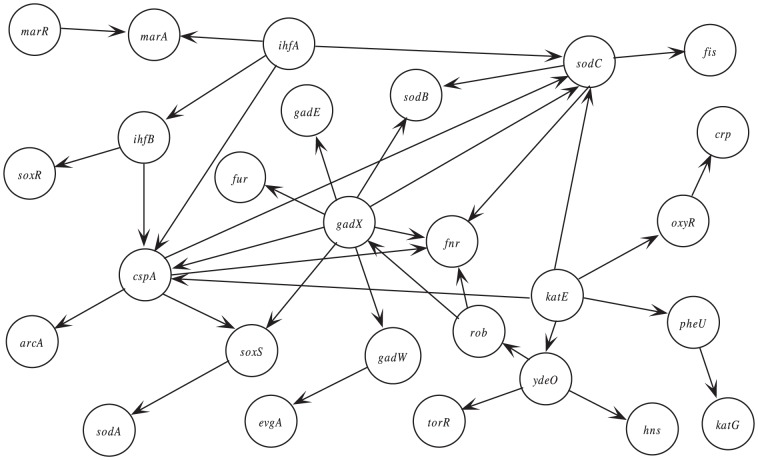
Consensus Bayesian network 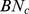. 27 genes were identified from the EcoCyc ROS detoxification pathway.

The consensus Bayesian network is constructed by combining Bayesian networks derived from literatures and Bayesian networks learned from microarray data. First, relevant literatures were reviewed and a ROS pathway was derived from the literature wrote by Hodges et al.[20], denoted as 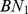. In the literature, 27 genes identified from the EcoCyc ROS detoxification pathway were chosen as variables and 305 microarray expressions were used to learn the Bayesian network. Second, microarray data was searched and a microarray expression build with 612 microarray expressions was downloaded from the M3D database. Then Bayesian network 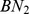 which also uses the 27 genes as variables was learned from these microarray expressions. Finally, consensus Bayesian network 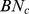 was constructed by combining these two Bayesian networks, and the result is shown in Figure 1. In the combination program, we take weights 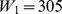, 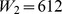 and threshold 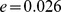.

A novel prediction algorithm based on the computation of mutual information was developed to identify genes which are strongly associated with a particular gene in the regulatory pathway. If 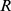 is a gene in the regulatory pathway, gene 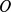 is strongly associated with 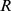, then 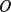 may work together with 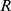 and also be involved in the pathway. The main procedure of this algorithm can be described as follow: assume set 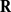 includes all the known genes in the regulatory pathway, and set 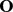 includes the rest genes of the organism. Choose one gene 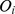 in 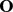, for each gene 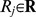, compute the mutual information 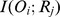, if 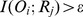, it means gene 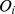 is related to gene 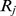, then 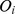 may be involved in the pathway too. The program is ended until every gene in 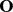 has been tested.

**Figure 2:**
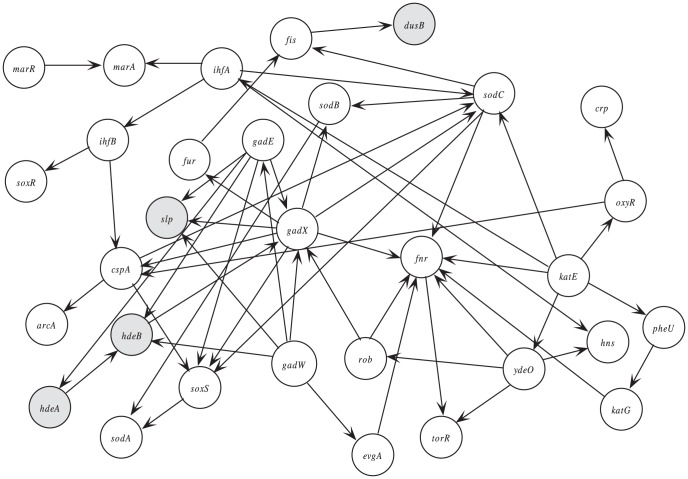
Consensus Bayesian network 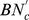. 27 genes were identified from the EcoCyc ROS detoxification pathway, while genes 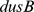, 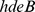, 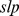, 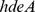 were identified by the prediction program.

27 genes identified from the EcoCyc ROS detoxification pathway were used as set 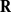, while the rest genes in 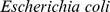 were used as set 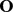. The program found 4 genes may be involved in the ROS pathway, and the results are shown in Table 3. A new Bayesian network 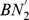 using the 31 (27+4) genes as variables was learned from the 612 microarray expressions. 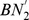 contains more genes than 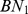, so 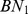 was extended into 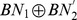. Then a new consensus Bayesian network 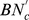 was constructed by combining 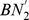 and 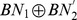, and the result is shown in Figure 2.

**Table 3:** 4 genes identified by the prediction program.

Gene 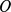	Gene 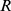	Mutual information 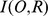
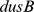	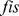	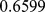
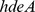	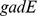	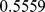
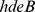	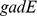	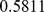
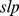	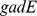	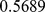

Genes 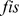, 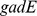 were identified from the EcoCyc ROS detoxification pathway. The interactions between gene 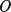 and gene 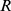 can also be found in EcoCyc database.

## Discussion

In the discussion, we address this question: does the Bayesian network learned from microarray expressions match with a known regulatory pathway?

Before answering this question, we carried out an experiment. The procedure of the experiment can be described as follow: assume that 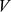 includes all of the genes of 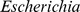 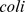, and then we construct an undirected graph 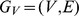, where 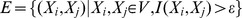. Let 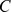 be the largest connected subgraph of 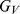. Then 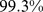 of the genes in 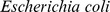 were included in 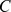. Mutual information 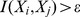 means genes 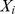 and 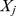 are interacted, so this phenomenon shows that almost all genes in 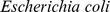 are related directly or indirectly. We can infer that some genes may be involved in different regulatory pathways, simultaneously. Otherwise, if there is no gene be involved in more than one regulatory pathway, that is, the regulatory pathways in 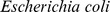 have no intersection, then we can't observe the phenomenon that thousands of genes related directly or indirectly. On the other hand, before microarray measurements, the 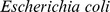 was alive, so almost all of the regulatory pathways of 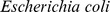 were at work. Then although two genes must be interacted if there is a directed edge between them in the Bayesian network, it is hard to determine the directed edge belongs to which regulatory pathway. For example, there is a directed edge between 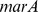 and 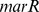 in 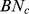 (Figure 1), then there must be an interaction between 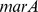 and 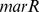. They are involved in the regulation of transcription (EcoCyc database) and this biological process was working when measuring the expressions of these genes using microarray, therefore, the existence of 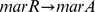 maybe due to that they are regulating the transcription. However, the ROS detoxification pathway (EcoCyc database) also contains 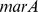 and 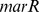, then the existence of 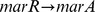 maybe due to that they are regulating the response to the oxidative stress. So, it is hard to determine the directed edge 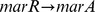 belongs to which regulatory pathway. If there is no edge between two genes in the Bayesian network, then the two genes are not interacted directly in any regulatory pathway. So, if a known regulatory pathway contains 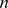 genes and we use these 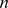 genes as variables to learn a Bayesian network from microarray expressions. Then all of the interactions between the 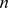 genes are contained in the Bayesian network, however, some of these interactions may not contained in this regulatory pathway. This means the regulatory pathway is a subgraph of the Bayesian network. Although the Bayesian network is not equivalent to the regulatory pathway, it still has important significance. With its guidance, the number of biological experiments could be greatly reduced when modeling a regulatory pathway.

## Methods

### Data preprocessing

The algorithms can only process discrete data in this paper. However, the 612 microarray expression data of 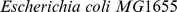 downloaded from the M3D database are continuous. Then expression data for each gene was discretized using a maximum entropy approach which uses three equally-sized bins (q3 quantization). And the genes' expressions were divided into three categories: underexpressed, normal, overexpressed.

Usually, Bayesian networks derived from literatures only have a structure, then we have three ways to obtain the parameters: (1) If the program of the learning algorithm is available on the internet, then both the structure and the parameters of the Bayesian network can be obtained by run the program directly. (2) If the microarray data used in the literatures were collected in a database available on the internet, then we can download these microarray data to learn the parameters. (3) Sometimes the corresponding database is unable to be found, or the Bayesian network is not learned form database, but constructed by biological experiments directly. Then distribution for each node can be estimated by analyzing the genes' special characteristics and the relationships between genes.

### Bayesian network

**Figure 3:**
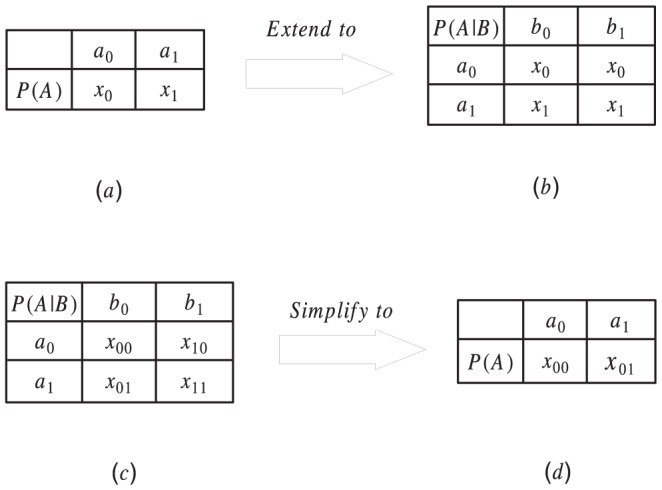
Extension and simplification of CPT.

A Bayesian network defined over a variable set 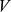 can be represented as a pair 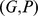, where 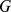 is a DAG and each directed edge in the DAG represents a dependence, 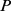 is a group of CPTs and each node in the DAG has a CPT. Usually, 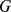 is called Bayesian network's structure and can be represented as a pair 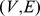, where 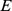 is the edge set; 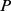 is called Bayesian network's parameter. 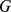 is a directed acyclic graph, that is, the nodes in 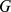 have a topological order, and we call it prior order. Let 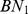 and 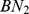 be two Bayesian networks and their DAGs are 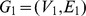 and 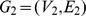, respectively, then 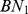 and 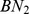
's variables' prior orders are consistent with each other if 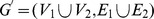 is acyclic. Let 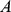 be a node in 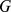, 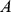
's direct precursor nodes are called its parents, denoted as 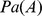, then 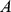
's CPT represents the conditional probability 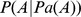. Suppose we have the CPT of 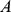 as shown in Figure 3(c), it shows 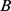 is a parent of 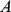 and 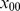 means 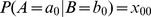. Assume that CPT 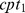 represents 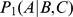, 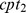 represents 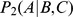 and 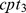 represents 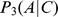. Then 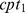 and 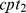 are two tables with the same structure and the conditional probabilities in the corresponding positions of the two tables represents the same conditional probability, so we say they have a same form. While 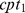 and 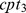 do not have a same form.

### Bayesian network learning algorithm

Usually, Bayesian network is learned from database, it represents the probabilistic relationships between the variables in the database. So, a Bayesian network matches with a database, and we call this database Bayesian network's corresponding database. Bayesian network learning includes structure learning and parameter learning. We use an information-theory based learning algorithm proposed by Cheng et al. [11] to learn Bayesian network's structure in this paper.

Dependence between two variables can be quantitatively computed by using mutual information. Mutual information 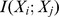 between two variables 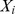 and 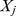 can be defined as:
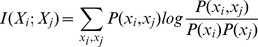
(1)


where 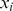, 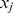 are the expression values of 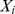 and 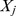, respectively. Mutual information is non-negative, it means 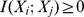. 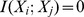 holds if and only if 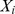 and 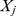 are independent. Given a threshold 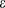 (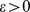), 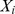 and 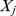 are related if 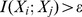. Similarly, conditional mutual information 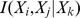 can be defined as:
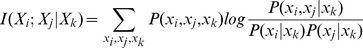
(2)


Then the main procedure of Cheng's Bayesian network structure learning algorithm can be described as follow:

Step 1. Create initial undirected graph. A Maximum Weight Span Tree (MWST) [23] is used as the initial graph. Let 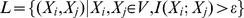 be an undirected edge list, where 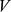 is the variable set. Sort 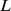 in descending order of mutual information. For each 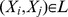, add it into the undirected graph(and delete it from 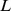) if it doesn't form a circle. End this loop until the graph contains 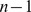 edges.

Step 2. Add edges. Assume that set 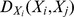 contains all the nodes which are in the paths between 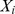 and 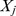 and in the neighborhood of 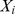, simultaneously. 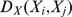 represents one of sets 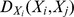 and 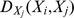 which contains less nodes. For each 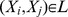, add it into the undirected graph(and delete it from 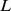) if 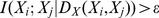 holds.

Step 3. Remove redundant edges. For each edge 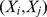 in the undirected graph, delete it if 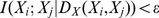 holds.

Step 4. Determine edges' directions. For each 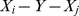, direct them 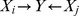 if
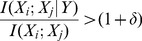
(3)


holds, where threshold 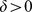. Some undirected edges' directions can be determined by using Bayesian network's acyclic property. For the rest undirected edges, use the local Minimal Description Length (MDL) score [24] to choose the direction which makes the MDL score more smaller.

In Bayesian network parameter learning, the following equation is used to compute the conditional probabilities in each node's CPT:
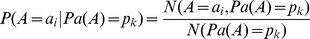
(4)


where 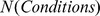 is the number of samples satisfies 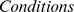 in the database.

### Extension and simplification of CPT

#### Theorem 1

Given variables 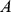 and 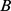, then 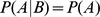 (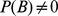) holds if 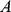 and 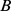 are independent.

#### Corollary 1

Given 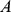, 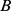 and any other variable 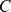, then 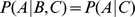 (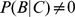) holds if 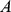 and 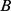 are independent given 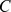.

**Figure 4:**
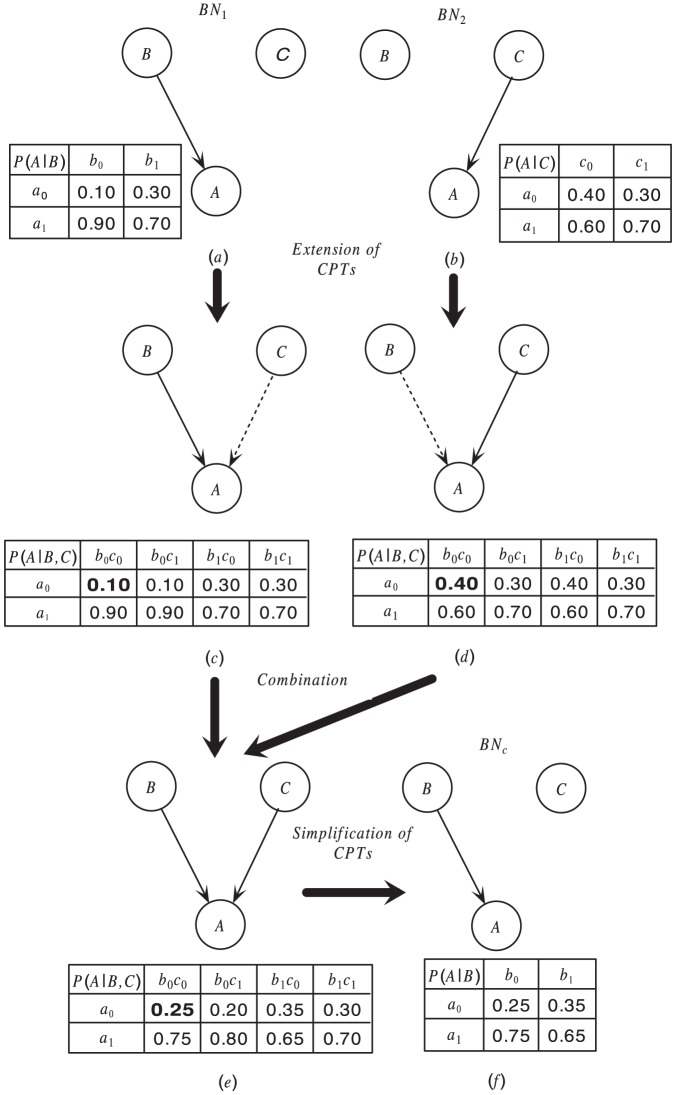
An example to demonstrate the combination of two Bayesian networks. Assume that weights 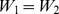, 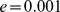, and we have two Bayesian networks as shown in 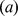 and 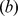. The CPTs of 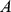 in the two Bayesian networks do not have a same form, so they need to be extended. After extending the CPTs, the two Bayesian networks' structures and every corresponding CPTs' forms are completely the same(as shown in 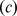 and 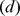, the dashed edges represent bogus edges), and then the aggregation function can be applied to aggregate the conditional probabilities in corresponding positions of each corresponding CPTs. For example, 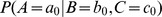 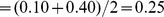. In the combined Bayesian network as shown in 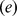, we need to use variance to test 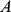
's two parent nodes, 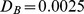 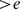, 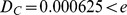, so 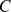 is a bogus parent. Then the CPT of 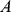 need to be simplified and the bogus edge 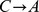 should be deleted. The consensus Bayesian network is shown in 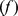.

Suppose we have the CPT of node 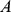 as shown in Figure 3(a), it can be extended into the form as shown in Figure 3(b) if 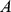 and 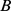 are independent of each other. Since 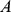 and 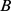 are independent, 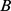 can not affect the distribution of 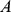, then for 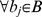, 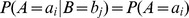 holds. According to that, two CPTs of a same node in different Bayesian networks can be extended into a same form, and then can be aggregated even if the node does not have a same parent set in these Bayesian networks. Specifically, for a node 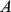, and its parent sets are 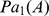 and 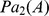 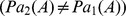 in 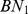 and 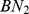, respectively. Then the two CPTs of 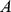 do not have a same form, and the aggregation function can't be applied (See the CPTs of 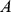 shown in Figure 3(b) and Figure 3(c), they have a same form, then the aggregation function can be applied to aggregate the conditional probabilities in the corresponding position of the two CPTs, and the aggregation function can't be applied to aggregate the CPTs shown in Figure 3(a) and Figure 3(c)). However, we can take 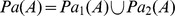 as the parent set and extend both the CPTs of 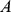 in 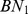 and 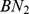 into form 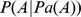, then the two CPTs of 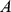 have a same form and the aggregation function can be applied. This means we also view the nodes in 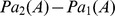 as the parents of 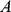 in 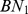, although they are not real parents and do not affect 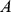
's conditional probability. We call these parents bogus parents and the directed edges between a node and its bogus parents bogus edges. As shown in Figure 4(c), 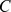 is a bogus parent of 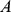, and 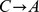 is a bogus edge.

#### Theorem 2

Given variables 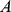 and 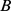, 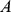 is independent of 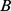 if the conditional probability of 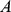 does not change when 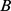 takes different values.

#### Proof

Assume that the number of expression values of 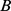 is 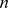 and for 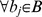, 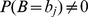. Then for 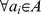, we have:
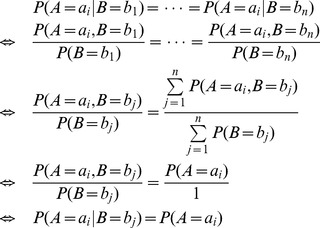



So, 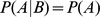, then 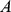 and 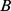 are independent.

End of the proof.

#### Corollary 2

Given 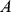, 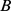 and any other variable 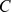, 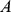 is independent of 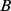 given 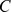 if the conditional probability of 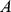 does not change when 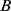 takes different values (only 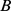 changes).

Theorem 2 and Corollary 2 can be used to determine whether two nodes are independent of each other or not. Suppose we have the CPT of node 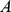 as shown in Figure 3(c), if 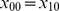, 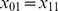 or they are approximately equal, it deduces that 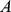 and 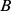 are independent, and 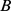 is not the parent node of 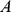. Then the CPT of node 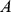 can be simplified into the form as shown in Figure 3(d). Conditional probabilities in the CPT of 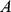 are discrete values, then variance can be used to determine whether the conditional probability of 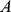 changes or not when 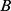 takes different values. Assume that 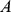
's parent set is 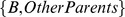. First, compute each variance 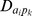 of the conditional probabilities satisfy 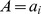, 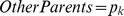 and 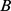 takes different values. Second, compute the average variance 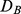 of all 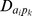 when 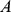 and 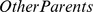 take different values. Given a threshold 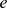 (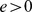), if 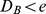, it means the conditional probability of 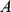 almost does not change when 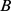 takes different values, then 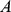 and 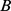 are independent and 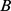 is not the parent node of 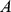. In the combination algorithm, CPTs were extended previously, then some nodes may have bogus parents after the aggregation of CPTs. However, we can use this method to find them, and then simplify the CPTs and delete the bogus edges. Threshold 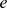 can be selected by using domain knowledge. Specifically, we have 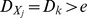 if variables 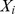 and 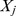 are related, and 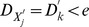 if variables 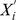 and 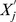 are independent. And then we have 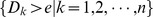 if there is 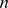 pair of variables related, and 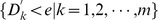 if there is 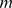 pair of variables independent each other. So we have 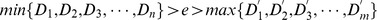.

### Aggregation function

Assume that the conditional probability of node 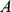 in Bayesian networks 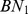 and 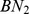 are 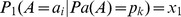 and 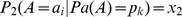, respectively. Then in the consensus Bayesian network, the conditional probability of 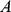 can be computed using the following equation:
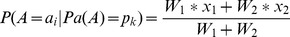
(5)


where 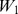 is the weight of 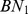 and 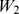 is the weight of 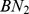. Weight 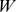 is a positive integer representing a belief to the Bayesian network. 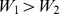 means 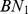 is more reliable than 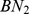; 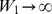 means 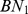 is absolutely reliable.

Next, we would like to discuss why we choose this aggregation function. The combination of Bayesian networks must satisfies this property: the consensus Bayesian network 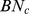 constructed by combining Bayesian networks 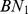 and 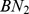 is equivalent to the Bayesian network learned from the database obtained by merging the two Bayesian networks' corresponding databases 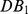 and 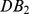. Then the aggregation function should satisfy it too. Assume that node 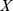 is not the parent of 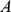 in any Bayesian network, then in the consensus Bayesian network, 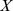 can not be the parent of 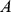. Then CPTs of 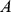 in different Bayesian networks after extension not only have a same form, but also contains all of 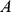
's possible parent nodes. So, we needn't consider the nodes which are not included in the parent set of 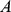 when aggregating the CPTs. When computing the conditional probability of one node in Bayesian network, Equation (4) is used. The conditional probability of 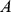 in Bayesian networks 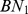 and 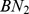 are 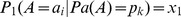 and 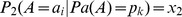, respectively. Assume that the number of samples satisfy 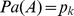 in 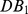 is 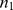, then the number of samples satisfy 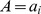 and 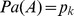 in 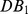 is 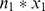; the number of samples satisfy 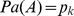 in 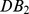 is 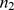, then the number of samples satisfy 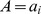 and 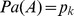 in 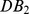 is 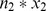. So, the conditional probability of 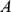 in the Bayesian network learned from the database obtained by merging 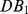 and 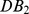 is:
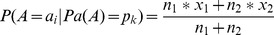
(6)


On the other hand, samples satisfy 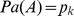 in 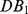 and in 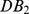 obey the same distribution. So, we have:
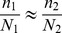
(7)


where 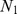 and 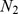 are the total numbers of samples in 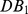 and 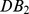, respectively. Then the conditional probability of 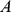 changed to be:
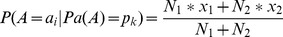
(8)


Total numbers of samples in databases are unable to be known sometimes, so we use the weights of the Bayesian networks instead of them, then Equation (8) changed into Equation (5). In the experiment, we still use the total numbers of samples as they are already known.

### Combination of Bayesian networks

If two Bayesian networks are defined over the same variable set and their variables' prior orders are consistent with each other, then they can be combined using the method described as follow:

Step 1. Extend every corresponding CPTs in the two Bayesian networks into same form. Then the structures of the two Bayesian networks are completely the same(although some of their edges are bogus edges).

Step 2. Use the aggregation function to aggregate the conditional probabilities in the corresponding positions of each corresponding CPTs.

Step 3. In each CPT after aggregation, compute variance 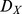 for each parent node 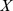, determine whether 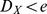 holds or not to judge node 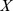 is a bogus parent or not, then simplify the CPT and delete the bogus edge if 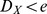 holds.

After simplifying the CPTs and deleting the bogus edges, the consensus Bayesian network is obtained. Figure 4 shows an example of combination of two Bayesian networks. However, Bayesian networks' variables' prior orders do not always consistent with each other, then it needs to reverse some directed edges sometimes. The principle of reversal is to ensure that the Bayesian network after reversal is equivalent to the original Bayesian network.

### Extension of Bayesian network

Sometimes the Bayesian networks going to be combined may not defined over the same variable set, then they need to be extended. Specifically, given two Bayesian networks 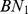 and 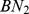 with their variable sets satisfy 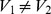 and 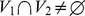, if their variables' prior orders are consistent with each other, 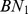 can be extended into 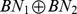 using the method described as follow:

Step 1. Extend 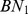
's DAG 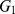 into 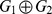. Let 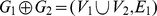, and then add all the directed edges satisfy
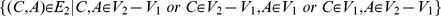



into graph 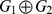. These added edges are not in 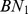 originally, so we call them extended edges.

**Figure 5:**
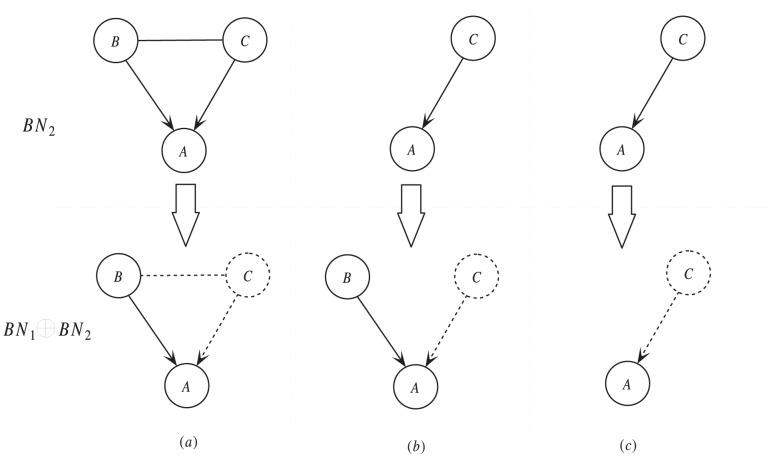
Three possible situations in the extended Bayesian network 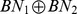. Where 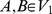, 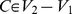, 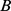 and 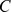 may be two nodes or two node sets with each node in them has a directed edge point to 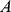. In 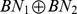, solid lines represent the edges in 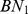 originally, and dashed lines represent the extended edges. The undirected edge 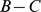 represents one of these three cases: (1) directed edge 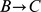; (2) directed edge 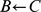; (3) 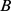 and 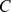 is disconnect.

Step 2. Compute each node's CPT. For a node 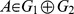, if 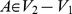, then its CPT is the same as the CPT of 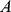 in 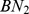; if 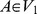 and there is no directed edge satisfies 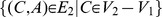, then its CPT is the same as the CPT of 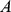 in 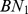; if 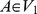 and has directed edges satisfy 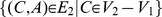, in this case, there are three possible situations may appeared in the extended Bayesian network as shown in Figure 5. Then the conditional probabilities of 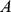 in these three situations can be computed using the following equations, respectively:

In Figure 5(a)

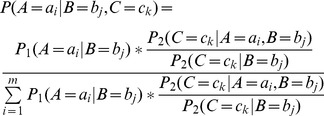
(9)


where 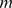 is the number of expression values of 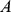. 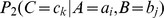 and 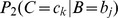 can be computed using the standard Bayesian network inference algorithm [25] in 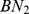, while 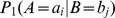 is already known in 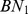.

In Figure 5(b)

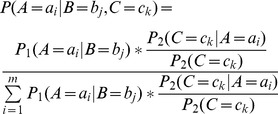
(10)


In Figure 5(c)

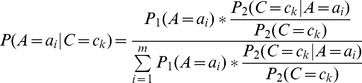
(11)


If 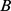 and 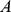 are disconnect in 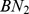, it can only deduce that 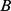 and 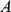 are independent given 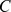, however, it doesn't affect the conditional probabilities 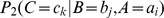 and 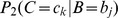, then the conditional probability of 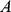 can be computed using Equation (9). If both 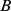 and 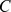, 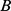 and 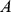 are disconnect in 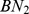, it deduces 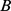 and 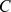 are independent, then the conditional probability of 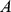 can be computed using Equation (10). After obtaining every node's CPT in 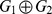, the extension of Bayesian network 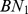 is finished.

After extending 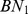 into 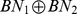 and 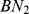 into 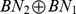, 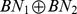 and 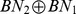 are defined over the same variable set 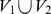, and then they can be combined using the combination algorithm.
