# Correction: Using Consensus Bayesian Network to Model the Reactive Oxygen Species Regulatory Pathway

**DOI:** 10.1371/annotation/4bf3c893-ad02-4730-b775-fd83fd8d174c

**Published:** 2013-08-13

**Authors:** Liangdong Hu, Limin Wang

A funding organization and grant were incorrectly omitted from the Funding Statement. The funding organization and grant are: National Natural Science Foundation of China (No. 61272209). 

